# Genome-Wide Identification and Characterization of GRAS Transcription Factor Family in Cultivated Hybrid Sugarcane ZZ1 (*Saccharum officinarum*) and Their Role in Development and Stress

**DOI:** 10.3390/ijms252413470

**Published:** 2024-12-16

**Authors:** Hao Wen, Lidan Wang, Yuqing Gong, Yu Zhang, Tingting Zhao, Cuilian Feng, Jungang Wang, Jishan Lin

**Affiliations:** 1 National Key Laboratory for Tropical Crop Breeding, Institute of Tropical Bioscience and Biotechnology, Chinese Academy of Tropical Agricultural Sciences, Sanya 572024, China; wenhaohy34@163.com (H.W.); zhaotingting@itbb.org.cn (T.Z.); fengcuilian@itbb.org.cn (C.F.); 2 School of Breeding and Multiplication (Sanya Institute of Breeding and Multiplication), Hainan University, Sanya 572025, China; 15528556909@163.com; 3 Haixia Institute of Science and Technology, Fujian Agriculture and Forestry University, Fuzhou 350002, China; gongyuqing497@163.com (Y.G.); 18055789001@163.com (Y.Z.)

**Keywords:** GRAS transcription factors, sugarcane hybrids (ZZ1), structural diversity, stress response, smut

## Abstract

GRAS gene family plays multifunctional roles in plant growth, development, and resistance to various biotic and abiotic stresses, belonging to the plant-specific transcription factor (TF) family. In this study, a genome-wide survey and systematic analysis of the GRAS family in cultivated hybrid sugarcane ZZ1 (*Saccharum officinarum*) with economic and industrial importance was carried out. We identified 747 GRAS genes with complete structural domains and classified these into 11 subfamilies by phylogenetic analyses, exhibiting a diverse range of molecular weight and isoelectric points, thereby indicating a broad structural and functional spectrum. Analysis of Protein motif and gene structure revealed a conserved yet variable arrangement of motifs within the GRAS TFs, suggesting its potential for diverse functional roles. Furthermore, the identification of numerous cis-regulatory elements by GRAS TFs promoter sequence analysis, implying their complex regulation in response to environmental and physiological signals. Tertiary structure predictions analyses using AlphaFold3 highlighted the structural flexibility and conservation within the GRAS family, with disordered regions potentially contributing to their functional versatility. Weighted Gene Co-expression Network Analysis (WGCNA) provided insights into the potential roles of *ShGRAS21A* in sugarcane’s response to smut infection. This comprehensive investigation of the GRAS family in ZZ1 not only uncovers their structural diversity but also sheds light on their potential regulatory roles in plant growth, development, and stress response. The findings contribute to a deeper understanding of GRAS TFs functions and lay the groundwork for future studies on their role in sugarcane improvement and disease resistance.

## 1. Introduction

Transcription factors (TFs) are proteins that specifically bind to nucleotide sequences located upstream of genes, thereby regulating the transcription of these genes [1,2]. Since the identification of the first transcription factor in maize, numerous TFs have been demonstrated to participate in various physiological processes and regulatory networks in higher plants [3,4]. As transcription factors, GRAS TFs are involved in various plant life activities, such as growth and development, stress response, and signal transduction [5,6].

The GRAS originated from three of the earliest characterized members: GAI (gibberellic acid insensitive), RGA (repressor of ga1-3), and SCR (scarecrow). Consequently, GRAS TFs are named after these three initial members [7,8,9,10]. In general, GRAS TFs possess a relatively conserved C-terminal domain that includes five highly conserved motifs: LRI (Leucine-rich regions I), VHIID, LRII (Leucine-rich regions II), PFYRE, and SAW, three of which (VHIID, PFYRE and SAW) were named after the conserved amino acid motifs [11]. This conserved C-terminal domain, along with a variable N-terminal domain, constitutes the fundamental structure of GRAS TFs [12,13]. The VHIID, PFYRE, and SAW are characterized as short or scattered conserved sequence elements [14]. Notably, the VHIID region, situated between LRI and LRII, is a conserved domain found in nearly all GRAS TFs [15,16]. Previous studies have suggested that the LRI-VHIID-LRII pattern may facilitate the binding of proteins to nucleic acids or the interaction with other proteins [17]. The GRAS family encompasses several subfamilies that exhibit both similarities and differences in their protein sequences. Early classifications of GRAS TFs in *Arabidopsis* and rice identified eight subfamilies: DELLA, LAS (LATERAL SUPPRESSOR), SCR, SHR (SHORT ROOT), PAT1 (PHYTOCHROME A SIGNAL TRANSDUCTION), HAM (HAIRY MERIST EM), SCL9 (LISCL; Lilium longiflorum SCR-like), and SCL4/7 [6]. To date, numerous GRAS TFs have been identified across various eukaryotic species, including 32 in Arabidopsis, 57 in rice [15], 53 in tomato [3], 62 in Barley [18], 48 in Chinses cabbage [19], 123 in Miscanthus sinensis [20], 73 in bananas [21], 45 in Pitaya [22], 50 in sweet orange [23], 48 in litchi [24], and 81 in sorghum [6].

Sugarcane (*Saccharum* spp., Poaceae) is an essential crop with significant economic value, contributing over 80% of the world’s sugar production and approximately 40% of bioethanol yield [25], which is estimated to have an annual economic value of up to $90 billion (https://www.fao.org (accessed on 15 August 2024)). Sugarcane is a polyploid interspecific hybrid, resulting from interspecific crosses with a complex genetic basis, leading to a large and complex genome and variable chromosome numbers [26]. Modern sugarcane hybrids (Sh) (ZZ1) originate from interspecific crosses between high-sugar *Saccharum officinarum* and the low-sugar *Saccharum spontaneum*, supplemented by extensive backcrossing with *S. officinarum* [27]. This hybridization process not only enhanced the vigor, robustness, tillering, disease resistance, and environmental adaptability of modern cultivated sugarcane hybrids but also increased the genomic complexity beyond that of their progenitors [27]. The resulting hybrid genome comprises a mix of aneuploid and homoeologous chromosomes, which are inherited unevenly from the two polyploid progenitor species, resulting in a substantial genome size of approximately 10 Gb. The chromosome number in these hybrids can vary from 100 to 130, with ZZ1 containing 114 chromosomes. Approximately 70 to 80% of these chromosomes are derived from *S. officinarum*, 10 to 20% from *S. spontaneum*, and around 10% arise from interspecific recombination [27]. The specific role of GRAS family genes in ZZ1 remains poorly understood. In this study, we performed a genome-wide survey to identify GRAS TFs in the ZZ1 genome. A systematic analysis was performed, including gene classification, characterization, structures analysis and phylogenetic relationships. The findings from this genetic investigation offer new insights and perspectives, providing insight into the GRAS genes in ZZ1 and laying the foundation for further research into the functional and evolutionary aspects of the GRAS family in plants.

## 2. Results

### 2.1. Identification and Localization of the GRAS TFs

Through a comprehensive genome-wide analysis of ZZ1, we identified 789 candidate genes belonging to the GRAS family. To refine our findings, structural domain annotations were meticulously revised to eliminate incorrectly predicted and redundant sequences. This process resulted in the identification of 747 GRAS transcription factors (TFs) that possess complete domains, characterized by the presence of at least three conserved GRAS structural motifs.

The ZZ1 genome was systematically partitioned into twelve distinct haplotype genomes, labeled from singleA to singleL, along with an additional contig genome, referred to as singleCtg (Table S1), based on their chromosomal origins [27]. These thirteen haplotype genomes contain 48, 33, 51, 50, 37, 59, 33, 54, 56, 69, 95, 41, and 121 members of GRAS family, respectively (Table S2). On average, each haplotype genome comprises 57 GRAS TFs, a count comparable to that found in other known species. This average exceeds the numbers observed in *Arabidopsis*, rice, tomato, Chinese cabbage, Pitaya, sweet orange, and litchi, yet remains lower than that in barley, *Miscanthus sinensis*, and *Sorghum*.

Subsequently, these transcription factors were systematically renamed as *ShGRAS1A* through *ShGRAS127Ctg* (Table S3), following the haplotype genome designation and their ascending chromosomal coordinates. In light of the polyploid nature of the organism, the corresponding alleles were delineated, resulting in the identification of 51 genes representing 747 alleles (Table S4). Localization analysis uncovered that the GRAS transcription factors were randomly distributed across both homologous and non-homologous chromosomes (Figures S1 and S2).

### 2.2. Molecular Weight, Isoelectric Point

To gain insights into the structural and functional properties of the GRAS TFs, we examined various characteristics, including gene length, protein sequence length, protein molecular weight (MW), and isoelectric point (pI) (Table S3). Our analysis revealed significant variations in length among the GRAS TFs (Table S3). Among the 747 identified ShGRAS TFs, *ShGRAS15I* was the largest, consisting of 1286 amino acids, whereas *ShGRAS9K* was the smallest, containing only 154 amino acids. The molecular weights of the proteins ranged from 18.183 kDa (*ShGRAS9K*) to 145.166 kDa (*ShGRAS62Ctg*), and the pI varied from 4.49 (*ShGRAS26A*) to 10.05 (*ShGRAS31F*), with a mean value of 6.28 (Table S3).

### 2.3. Phylogenetic and Clade Analyses

**Figure 1:**
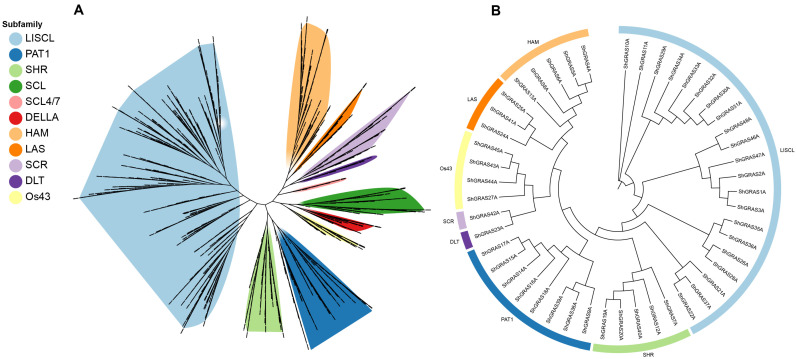
(**A**) Unrooted phylogenetic tree showing relationships among GRAS domains of ZZ1, *Arabidopsis thaliana*, *Oryza sativa*, *Sorghum bicolor*, and *Miscanthus sinensis*. (**B**) Phylogenetic tree constructed by maximum likelihood (ML) estimation of the GRAS TFs in the singleA genome of ZZ1. Different colors were used to classify subfamilies.

The 747 GRAS proteins identified in the ZZ1 genome, together with those from *Arabidopsis*, *Oryza sativa*, *Sorghum bicolor* and *Miscanthus sinensis*, were used to construct a maximum likelihood (ML) phylogenetic unroot tree (Figure 1A). This approach was adopted to systematically explore the intricate relationships within the ShGRAS family. These GRAS members were classified into 11 subfamilies based on established phylogenetic affiliations: LISCL, HAM, SHR, PAT1, DLT, LAS, DELLA, Os43, SCL, SCL4/7, and SCR. Predominantly, the GRAS members were concentrated within the LISCL, SHR, PAT1, and DELLA subfamilies, whereas DLT and SCL4/7 had the fewest members, averaging less than one per haplotype genome. The distribution of GRAS transcription factors across the various subfamilies in ZZ1 was as follows: LISCL (355), PAT1 (85), HAM (70), SHR (54), DELLA (24), Os43 (32), SCL (43), SCR (43), LAS (25), DLT (9), and SCL4/7 (7) (Table S5). Within the singleA haplotype genome, GRAS members were distributed across eight subfamilies, excluding DELLA, SCL, and SCL4/7 (Figure 1B). Significant variations were observed in the classification of GRAS transcription factors across different haplotypes (Figure S3).

### 2.4. Protein Motif and Gene Structure Analysis

**Figure 2:**
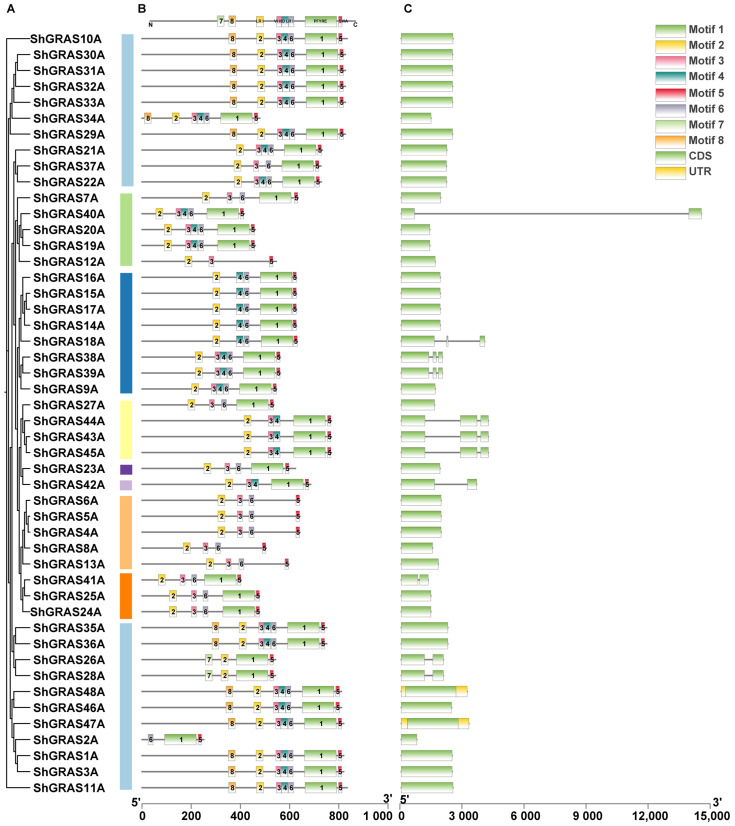
Phylogenetic relationships, motif distributions, and gene structure analysis of GRAS TFs in the singleA genome of ZZ1. (**A**) Phylogenetic trees were constructed using the maximum likelihood estimation method. (**B**) Amino acid motifs in the ShGRAS TFs (1–8) are represented by colored boxes. The black lines indicate relative protein lengths. (**C**) Introns are indicated by grey lines, green rectangles and yellow rectangles represent CDS and UTR, respectively.

To further explore the characteristics of the conserved domains of GRAS proteins in ZZ1, we performed a domains search using MEME for the ShGRAS TFs. Although our initial aim was to identify 10 motifs, only 8 were detected, reflecting a substantial degree of conservation within the GRAS protein sequences, with very few motifs present beyond their inherent motifs (Table S6). The SAW motif (motif 5) emerges as a relatively conserved C-terminal structural domain of GRAS transcription factors, ubiquitously present at the terminus of every GRAS protein (Figure 2B). Furthermore, the sequence arrangement of these identified motifs in GRAS transcription factors adheres to the following order: LR I (motif 2)—VHIID (motif 3)—LR II (motifs 4 and 6)—PFYRE (motif 1)—SAW (motif 5) (Figure 2B).

Notably, Motif 7 and 8 are exclusively found in the LISCL subfamily and are neatly located before LR I (motif 2). According to InterPro (https://www.ebi.ac.uk/interpro (accessed on 20 August 2024)), motif 7 and 8 do not correspond to established GRAS motifs and may be related to the functional roles of the LISCL subfamily of GRAS transcription factors, potentially serving as auxiliary domains or regulatory regions. In conclusion, GRAS TFs of ZZ1 exhibit a relatively complete motif structure overall, with only a few instances of one or two missing motifs (Figure S4). The structural analysis of the GRAS TFs in ZZ1 revealed that the members of the GRAS family possess a relatively conserved and simplified gene structure characterized by the absence of introns (Figure 2C and Figure S4). The member of exons varied from 1 to 11; only 145 genes (19.5%) contained multiple exons, while the 602 genes (80.5%) lacked introns entirely. Genes without introns exhibit a compact structure and are broadly distributed across all subfamilies.

### 2.5. Analysis of Cis-Regulatory Elements in Promoter Regions

**Figure 3:**
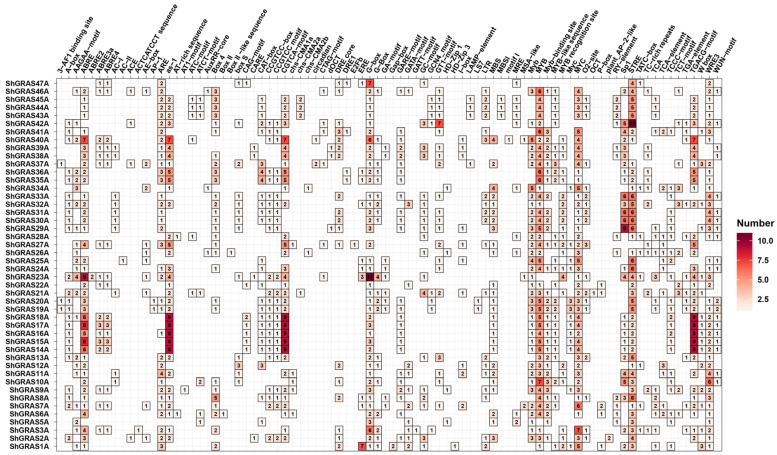
Analysis of cis-regulatory elements in the GRAS TFs promoter in the singleA genome. Dark red squares indicate more cis-regulatory elements, light red squares indicate fewer cis-regulatory elements, and white squares indicate no cis-regulatory elements.

To elucidate the cis-regulatory elements of GRAS TFs, we investigated the 2000 bp upstream promoter regions of the GRAS TFs in the singleA genome. This study aimed to reveal the complex array of cis-regulatory elements that modulate gene expression in response to various environmental and physiological signals. A total of 83 cis-regulatory elements were identified within the promoter sequence of the GRAS TFs, encompassing various physiological functions (Table S7). These elements were classified into seven categories: development-related, light-responsive, site-binding, environmental stress-responsive, hormone-responsive, wound-responsive, and other elements. Among these, eleven hormone-responsive elements were identified, including those responsive to abscisic acid (ABRE related), auxin (TGA-box), gibberellin (P-box, GARE-motif, TATC-box), MeJA (TGACG-motif, CGTCA-motif), and salicylic acid (TCA-element). Additionally, the investigation revealed a plethora of elements responsive to various light signals, including sp1, the 3-AF1 binding site, ACE, the AE-box, AT1-motif, ATC-motif, ATCT-motif, chs-CMA1a, Box-4, and others, illustrating the complexity of light-mediated gene regulation. The narrative of stress responsiveness was further enriched by the identification of elements such as the low-temperature responsive element (LTRE), the stress-responsive element (STRE), MYC, MYB, TC-rich repeats, and the W-box, each highlighting the resilience and adaptability of these species under stress conditions (Figure 3). Furthermore, it was observed that the majority of the identified cis-regulatory elements were enriched in MYB, MYC, sp1, STRE, and TGACG-motif.

### 2.6. Tertiary Structure

By conducting AlphaFold3 three-dimensional (3D) structural model predictions on the protein sequences of 11 GRAS subfamily members from the ZZ1 and 11 corresponding subfamily members from *Sorghum*, we generated a series of structural models. Subsequent visualization and analysis of these models using PyMOL (v3.1.1, https://www.pymol.org/) software revealed several notable structural features. In the prediction of tertiary structure, all ranking scores were above 0.8, indicating that the three-dimensional modeling of the different GRAS subfamily members was reliable (Table 1).

**Figure 4:**
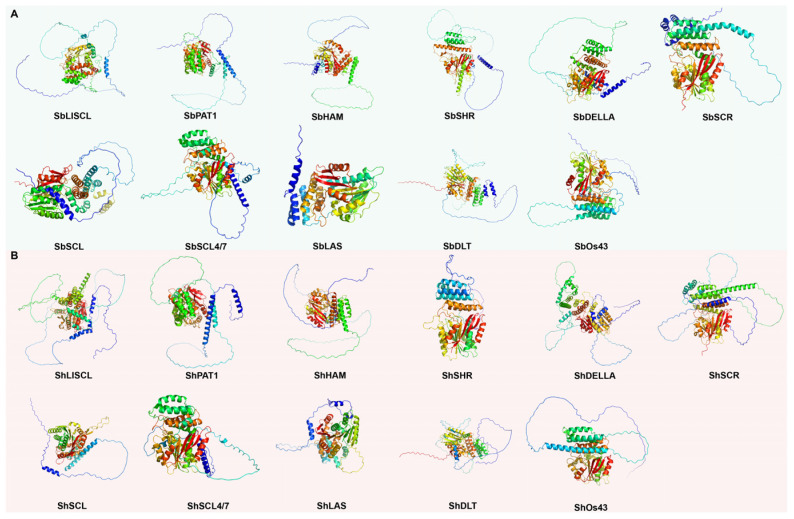
(**A**) Protein tertiary structure of representative member of 11 subfamilies of GRAS from sorghum. (**B**) Protein tertiary structure of representative member of 11 subfamilies of GRAS from ZZ1. Subfamilies are sorted by number of members in descending order.

In both sorghum and ZZ1, GRAS TFs exhibited, on average, 30% fractional disordered (Table 1, Figure 4). These disordered regions confer increased flexibility and dynamism to the GRAS TFs. Additionally, notable differences were observed among the GRAS subfamilies in ZZ1 and sorghum. Specifically, in sorghum, SbLAS has the lowest degree of disordered regions at only 5%, whereas in ZZ1, ShSHR exhibited the lowest degree at 13%. These differences illustrate the diversity of GRAS family members among different plants species and highlight various adaptations or functional divergence that have occurred throughout evolution. In addition to the observed similarities and differences between the disordered regions, the 3D structures of the proteins in the sorghum and ZZ1 subfamilies showed notable similarities, especially in the core structural domains.

Regarding root mean square deviation (RMSD), ShSCR, and SbSCR demonstrated the lowest RMSD value (0.226), indicating that these two proteins share the highest structural similarity. Conversely, ShDELLA, and SbDELLA had the highest RMSD value (28.276), indicating the greatest structural divergence between them. ShDLT and SbDLT had the highest matchalign score (3037.5), which is consistent with their low RMSD values, thereby reinforcing the notion of their structural similarity. In contrast, ShDELLA has a lower matchalign score (837.0) with SbDELLA, consistent with their high RMSD values, suggesting greater structural differences (Table 1, Figure 4).

### 2.7. Expression and Network Analysis

**Figure 5:**
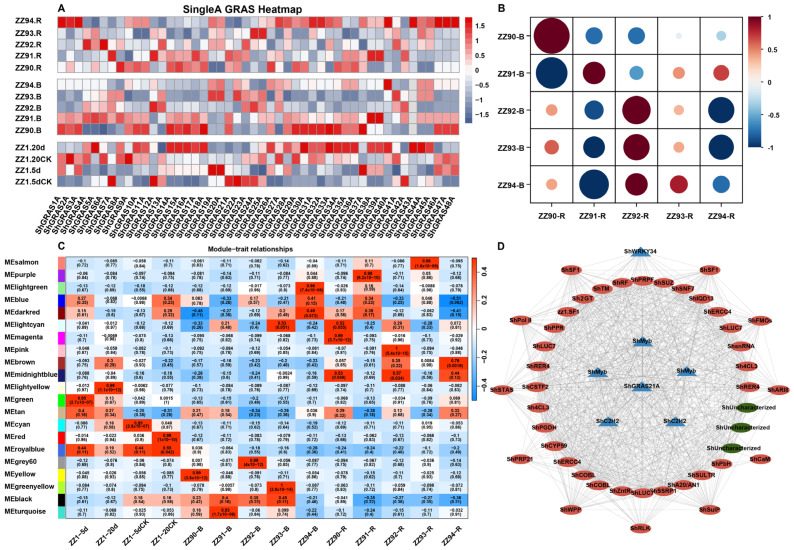
(**A**) Expression of GRAS TFs of ZZ1 in leaves (inoculation with Ssc spore treatment and water control) at 5 and 20 days and inoculated ZZ9 buds and roots, 0 to 4 days. (**B**) Correlation of GRAS TFs expression in buds and roots inoculated with ZZ9 between 0 and 4 days. (**C**) 21 expression profile modules identified by WGCNA. (**D**) Network diagram of the top 50 highly related genes centred on *ShGRAS21A*. From the inside out, the first and second circles are 5 transcription factors and 32 genes directly related by *ShGRAS21A*, and 12 genes in the outermost circle are genes and transcription factors indirectly related by *ShGRAS21A*. Triangles represent transcription factors and circles represent genes.

To elucidate the potential role of GRAS TFs in sugarcane smut, we examined the expression patterns of GRAS TFs within ZZ1 genome. The expression of GRAS TFs in samples from ZZ1 leaves inoculated with Ssc spores for 5 and 20 days, as well as those treated with water, exhibited diversity. Notably, genes in the PAT1 subfamily (*ShGRAS14A*, *ShGRAS15A*, *ShGRAS16A*, *ShGRAS17A*, *ShGRAS18A*, *ShGRAS38A*, *ShGRAS39A*), with the exception of *ShGRAS9A*, demonstrated increased expression following inoculation (Figure 5A). In contrast, certain genes showed reduced expression after Ssc spore inoculation, including *ShGRAS21A* and *ShGRAS22A* of the LISCL subfamily, *ShGRAS24A* and *ShGRAS25A* of the LAS subfamily, *ShGRAS23A* of the DLT subfamily, and *ShGRAS42A* of the SCR subfamily (Figure 5A). Furthermore, following the inoculating ZZ1 buds and roots, the expression of GRAS TFs displayed a significant positive correlation from day 0 to day 3, but a significant negative correlation by day 4 (Figure 5A,B).

WGCNA identified 21 modules of correlated expression, of which 14 had correlation coefficients exceeding 0.7 (*p* < 0.001) (Figure 5C). The MEblack module exhibited a distinct relationship, showing a positive correlation between leaf-treated and untreated samples, a negative correlation of expression between buds and roots, thereby categorizing it as a strongly differentiated module. The top 50 most highly associated genes within the MEblack module were extracted and functionally annotated (Table S8) to construct regulatory networks (Figure 5D). Among these 50 highly correlated genes, *ShGRAS21A* exhibited a direct related relationship with 37 genes, including C2H2 and MYB transcription factors, *ShCYP59*, *ShSULTR*, *ShRER4*, and other proteins associated with plant disease resistance and defense. Additionally, there are 12 genes indirectly related by *ShGRAS21A*, including *ShRLK*, *ShARI8*, *ShSulP*, *ShCaM*, *ShA20/AN1*, and *ShWRKY34*, which are implicated in stress responses potentially related to plant disease resistance. Collectively, these genes may play a role in response to smut infection in sugarcane.

### 2.8. Expression Pattern Analysis of ShGRASs by RT-qPCR

**Figure 6:**
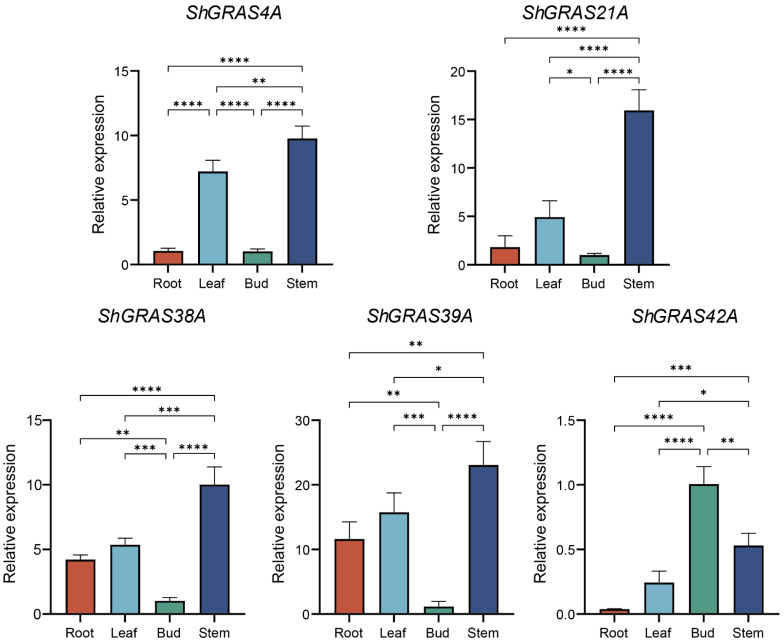
Expression profiles of different organs of ShGRAS by RT-qPCR. The relative expression levels of the target genes were determined using the 2^−ΔΔCt^ method. Asterisks indicate significant up-regulation of the corresponding genes (* *p* < 0.05, ** *p* < 0.01, *** *p* < 0.001, **** *p* < 0.0001).

To elucidate the expression of GRAS TFs across various organs in sugarcane, selected genes were analyzed by RT-qPCR and primers were designed using Primer 5 (Table S9). The findings indicate that, except for *ShGRAS42A*, these genes are more highly expressed in leaves and stems, with lower expression in roots and buds, particularly for *ShGRAS4A* and *ShGRAS21A* (Figure 6). Overall, the relative expression in stems were all higher, and the expression pattern showed a hierarchy of relative expression: stem > leaf > root > bud, and the relative expression in stems was almost 11.1 times higher than that in buds, about 7.3 times higher than that in roots, and about 2 times higher for leaves. The marked expression in leaves and stems suggests that they play a key role in these organs, and also indicates their importance in fundamental physiological processes such as growth regulation and stress response.

## 3. Discussion

Members of the GRAS transcription factor family exhibit a diverse array of functions and are pivotal in orchestrating various plant developmental and physiological processes. These include roles in gibberellin (GA) signal transduction, root and axillary shoot development, as well as transcriptional regulation in response to both abiotic and biotic stresses [28]. GRAS transcription factors have been identified and functionally characterized across a range of crops, revealing the molecular pathways through which these factors mediate plant responses to environmental stress. Despite these advances, a significant gap persists in correlation studies specifically related to cultivated sugarcane.

In this study, an extensive genome-wide analysis of the ZZ1 sugarcane variety led to the identification of 789 candidate genes within the GRAS family. After refining the structural domain annotations, 747 GRAS transcription factors with complete structural domains were confirmed. Due to the polyploid nature of the species, the ZZ1 genome was delineated into 12 haploid genomes and one Ctg genome that remains unassembled at the chromosomal level, and 51 genes were identified as representative of the 747 alleles, thus laying a solid foundation for future investigations into the GRAS transcription factor family in sugarcane. On average, there are 57 GRAS transcription factors per haplotype genome, a number comparable to the number of genes in some species. The observed variations in the GRAS transcription factor family among various plant species may be attributed to the differences in genome size or to gene duplication events that have occurred throughout evolutionary history [19].

Localization analyses demonstrated that GRAS transcription factors are randomly distributed across both homologous and non-homologous chromosomes, a pattern likely resulting from gene family expansion and recombination events. The length of the 747 ShGRAS proteins ranged from 154 to 1286 amino acids. Molecular weights spanned from 18.183 kDa to 145.166 kDa, and isoelectric points varied between 4.49 and 10.05. These findings underscore the considerable structural diversity of GRAS transcription factors, hinting at their potential to fulfill a variety of roles in sugarcane growth and environmental adaptation. Through the construction of a maximum likelihood (ML) phylogenetic tree, we categorized the GRAS transcription factors in ZZ1 into 11 distinct subfamilies. Among these, the LISCL, PAT1, HAM, and SHR subfamilies boasted the most members, whereas the DLT and SCL4/7 subfamilies had the fewest. This distribution suggests that each subfamily may have evolved to undertake specific roles in plant development and response to environmental stimuli.

By conducting a motif search for the GRAS transcription factors of the modern sugarcane hybrid ZZ1 using the MEME (v5.5.6) tools, we discerned that GRAS protein sequences are profoundly conserved, encompassing only eight major motifs. Notably, the SAW motif (motif 5) emerged as a relatively conserved C-terminal structural domain within the GRAS transcription factors. The five principal motifs of the GRAS transcription factors are sequentially ordered as LR I (motif 2)-VHIID (motif 3)-LR II (motifs 4 and 6)-PFYRE (motif 1)-SAW (motif 5), aligning closely with findings from prior studies. Moreover, motifs 7 and 8 were uniquely identified within the LISCL subfamily, suggesting a potential link to specific auxiliary functions or regulatory roles inherent to this subfamily. These two novel motifs warrant further verification and investigation.

The structural analysis of GRAS transcription factors in ZZ1 indicates that members of these gene families exhibit a relatively conserved and simplified gene structure, notably lacking introns. While the number of exons ranged from 1 to 11, a mere 19.5% of the genes (145) contained multiple exons, while the other 80.5% (602) being intronless genes (IGs), which is similar to the intronless structure of 84% in pepper [29], 77.4% in tomato [3], and 78% in switchgrass [30]. This compact gene architecture is consistently observed across all subfamilies. This phenomenon may be attributed to the ancestral origin of GRAS transcription factors in plants from ancient prokaryotes, reflecting their prokaryotic lineage followed by extensive duplication events throughout their evolutionary history, as genes in prokaryotic genomes are typically devoid of introns, and GRAS transcription factors share a close evolutionary relationship with such origins [3].

To reveal the cis-regulatory elements of the GRAS TFs, we analyzed the promoter region located 2000 bp upstream of the GRAS TFs in singleA of the ZZ1 genome. A total of 83 cis-regulatory elements were identified and classified into seven categories. Notably, among the cis-regulatory elements, a significant number of light-responsive elements were identified, such as sp1, 3-AF1 binding site, ACE and Box-4. These elements provide a complex picture of light-mediated gene regulation. Additionally, a substantial presence of stress-responsive elements, such as MYC, MYB and W-box, were observed, suggesting that GRAS provides higher adaptation and resilience to species under stress conditions.

We used AlphaFold3 to predict the three-dimensional structures of genes representing each subfamily of different GRAS TFs in ZZ1 and sorghum. All models achieved scores surpassing 0.8, attesting to their reliable 3D modeling quality. Analysis revealed that GRAS transcription factors in both ZZ1 and sorghum display, on average, approximately 30% partially disordered regions. The higher percentage of disordered regions implies that GRAS may possess enhanced structural flexibility, which is crucial for precise recognition and binding to their target sequences within the cell environment [31].

Intrinsically disordered proteins (IDPs) comprise a large fraction of eukaryotic proteomes and are important for cellular functions. Prevalent in cellular regulation, signaling networks, and disease pathways [32]. An IDR (intrinsically disordered region) within an IDP often undergoes disorder-to-order transitions upon binding to various partners, allowing an IDP to recognize and bind different partners at various binding interfaces [33]. Plant-specific GRAS TFs play critical and diverse roles in plant development and signalling, and act as integrators of signals from multiple plant growth regulatory and environmental inputs [31,34]. It suggests that sugarcane (ZZ1) GRAS transcription factors, as integrators of signals, may respond rapidly upon encountering pathogen invasion signals by regulating other genes involved in resistance to pathogens, while the IDR of GRAS will also play a role in recognizing pathogen signals or regulating defense responses, allowing them to adapt to different regulatory needs and environmental conditions. In summary, sugarcane GRAS transcription factors may be involved in the plant’s biotic and abiotic stresses, adapting to environmental cues and pathogen invasions by transitioning from a disordered to an ordered state, which may thereby modulate response to sugarcane smut disease.

As knockdown of *OsSCL7* resulted in decreased resistance to rice blast, whereas overexpression of *OsSCL7* increased resistance to rice blast [35], SLR1 gain-of-function mutants *SLR1-d1* and *SLR1-d2* showed increased resistance to *Magnaporthe oryzae* and *Xanthomonas oryzae* with increased resistance [36]. The transcription factor *OsGRAS30* and interaction with *OsHDAC1* increased histone H3K27ac levels, thereby enhancing broad-spectrum rice blast resistance [37]. It indicates that GRAS TFs play a role in plant disease resistance. A total of 21 correlated expression modules were identified by WGCNA. Notably, the MEblack module exhibited a unique expression pattern: it demonstrated a positive correlation between treated and untreated leaf samples, a negative correlation of expression between buds and roots, indicating a high degree of differentiation. A comprehensive regulatory network was constructed by extracting the 50 most connected genes within the MEblack module and annotating their functions. Among these genes, *ShGRAS21A* has a direct relate relationship with 37 genes, including C2H2 and MYB transcription factors, as well as *ShCYP59* [38], *ShZntR* [39], and *ShPPR* [40], among others. These genes are involved in plant disease resistance and defense. Furthermore, 12 genes were found to be indirectly related by *ShGRAS21A*, including *ShRLK* [41], *ShARI8* [42], *ShCaM* [43], *ShA20/AN1* [44], and *ShWRKY34* [45] among others, which are involved in stress response and plant disease resistance. These relationships suggest that this *ShGRAS21A* TFs may play a role in the regulation of disease resistance response in sugarcane.

RT-qPCR results showed that GRAS transcription factors were differently expressed in different organs of sugarcane, which implies that they are related to growth, adaptation [5,6], and even disease resistance of sugarcane. The expression of GRAS genes in stems was 11.1 times higher than that in shoots, 7.3 times higher than that in roots and 2 times higher than that in leaves. This suggests that they play a key role in the physiological processes of stems and leaves. The higher expression may be related to rapid growth or environmental sensitivity. The high expression of GRAS transcription factors in stems and leaves, as organs of sugarcane in direct contact with the external environment, may be related to their responsiveness to environmental changes. Similarly, they are the main targets of pathogen infestation, so the high expression of GRAS transcription factors in these organs may play a key role in disease resistance.

## 4. Conclusions

This study illuminates the structural diversity, distribution, and classification of the GRAS transcription factor family within the ZZ1 sugarcane genome through an exhaustive analysis. By thoroughly examining the motifs, gene structures, and three-dimensional structural characteristics of the GRAS transcription factors, we have uncovered essential insights into their functional roles in plant growth and environmental adaptation. The results of the WGCNA imply that GRAS transcription factors may hold a pivotal role in sugarcane’s response to smut disease. In particular, *ShGRAS21A* appears to be instrumental in orchestrating disease resistance responses and defense mechanisms in sugarcane, potentially through direct or indirect regulatory interactions with various genes associated with disease resistance and defense. These findings furnish theoretical support for the enhancement of sugarcane varieties and provide crucial insights into the specific mechanisms by which GRAS transcription factors confer disease resistance in sugarcane.

## 5. Materials and Methods

### 5.1. Data Resources

The sequences of the GRAS TFs in *Arabidopsis thaliana*, *Oryza sativa*, and *Sorghum bicolor* were acquired from the *Arabidopsis* Information Resource (TAIR) (https://www.arabidopsis.org (accessed on 10 August 2024)), the Rice Genome Annotation Project database (http://rice.uga.edu (accessed on 10 August 2024)), and articles about Sorghum GRAS [6], respectively. The genomic data about sugarcane hybrid ZZ1 were sourced from the China National Center for Bioinformation Genome Warehouse under accession code GWHEQVP00000000 [27]. RNA-seq raw data for ZZ1-related were obtained from NCBI under accession PRJNA1083323 [27].

### 5.2. GRAS TFs Identification and Chromosome Distribution

To identify the nonredundant GRAS TFs within ZZ1, we employed hidden Markov model (HMM) profiles corresponding to the GRAS domain (PF03514) downloaded from the InterPro database (https://www.ebi.ac.uk/interpro (accessed on 10 August 2024)). Using HMMER software, version 3.3.2 [46], searches were conducted against the proteomic sequences of the genomes with the default parameters, applying a stringent filter threshold of 0.01. Concurrently, sequence alignments were executed using the NCBI local BLAST tool (v2.16.0) [47], adopting an e-value threshold of 1 × 10^−5^, and sequences were filtered to include only those exhibiting an identity of more than 40%. To ensure the reliability of our findings, we further corroborated the derived sequences through the NCBI Conserved Domain Search Service (CD Search) (https://www.ncbi.nlm.nih.gov/cdd (accessed on 10 August 2024)), the Pfam database (http://pfam.sanger.ac.uk (accessed on 10 August 2024)), and the SMART tool (http://smart.embl-heidelberg.de (accessed on 10 August 2024)). These resources were instrumental in verifying the presence and completeness of the GRAS domain within our identified sequences. During the analysis, genes lacking the fundamental GRAS domain were meticulously screened. Additionally, genes misannotated due to aberrant length, either exceedingly long or short, were detected. Through this stringent protocol, we accurately identified the number of GRAS TFs in ZZ1.

Information on the physical location of each GRAS TFs on the genomic chromosome was obtained, and its localization was visualized by the R package karyoploteR 1.33.0 [48].

### 5.3. Molecular Weight, Isoelectric Point Analysis

To estimate the physicochemical parameters of each gene, including molecular weight (MW) and isoelectric point (pI), we employed the pI/Mw tool within the Peptides (v2.4.6) package [49]. Concurrently, we meticulously documented the chromosomal localization information of these genes.

### 5.4. Phylogenetic and Class Analyses

Using the MUSCLE algorithm [50], we conducted a multiple sequence alignment (MSA) of the full-length protein sequences of the GRAS family. Following the alignment, we utilized the RAxML software (v8.2.12) [51] to construct a phylogenetic tree using the maximum likelihood (ML) method. The resulting phylogenetic tree was then visualized and further edited using the iTOL platform (https://itol.embl.de (accessed on 12 August 2024)).

Using the classification system established for *Arabidopsis*, Rice, *Sorghum bicolor* and *Miscanthus sinensis* GRAS TFs [6,16,20,52], we systematically categorized the identified GRAS TFs of ZZ1.

### 5.5. Gene Structure and Conserved Motif Analysis

Introns and exons of each GRAS TFs on the singleA haplotype genome of ZZ1 were analyzed using TBtools v1.046 [53]. The conserved motifs within the GRAS protein family were explored with the aid of the motif elicitation (MEME) tool (v5.5.6, https://meme-suite.org/meme/tools/meme (accessed on 15 August 2024)) [54]. In our analysis, the parameter for the maximum number of motifs to be detected was set to 10. The motif size distribution was left to ‘any’. All remaining parameters were left at their default values, ensuring that the analysis was conducted with the standard settings recommended by the MEME suite. All results above were visualized by TBtools v1.046.

### 5.6. Promoter Analysis

The DNA sequences 2000 bp upstream of the initiation codon “ATG” of the GRAS TFs were defined as the promoter sequences. Subsequently, based on these inferred promoter sequences, we used the PlantCARE database [55] to predict the cis-regulatory elements that may govern the transcriptional regulation of these genes. The identification and quantification of these elements were conducted using R.

To ensure a rigorous analysis, we excluded unidentified elements and essential eukaryotic components such as the TATA-box and CAAT-box to concentrate on elements that possess specific regulatory functions.

### 5.7. Tertiary Structure Analysis

To determine the structural characteristics of the GRAS TFs, we randomly selected protein sequences representing each of the 11 subfamilies of modern sugarcane hybrids (ZZ1) and each of the 11 corresponding subfamilies of Sorghum. Subsequently, we used AlphaFold3 server (https://alphafoldserver.com (accessed on 17 August 2024)) [56] to generate three-dimensional structural models of these proteins. The resulting models were then visualized and analyzed using PyMOL software [57], which allowed us to present the predicted tertiary structures in a clear and accessible manner. The root means square difference (RMSD) and the MatchAlign score between the corresponding subfamilies were also calculated using PyMOL software.

### 5.8. Transcriptome and Expression Analysis

We downloaded the RNA-seq data for the roots and buds of ZZ9 (the same parents as ZZ1, derived from ROC25 and YZ89-7, which is highly resistant to smut), including ZZ90.R–ZZ94.R and ZZ90.B–ZZ94.B. The roots and buds were inoculated with Ssc spores (the causative agent of smut) (1 × 106 spores/mL) at 0 days, 1 day, 2 days, 3 days, and 4 days. Similarly, we also downloaded RNA-seq (ZZ1.5d and ZZ1.20d) samples collected from ZZ1 leaves inoculated with Ssc spore suspension at 5 days and 20 days post-inoculation, as well as samples treated with water as a control (ZZ1.5ck and ZZ1.20ck).

The GRAS family of the singleA haplotype of the ZZ1 genome was selected as the subject of the investigation. The downloaded RNA-seq data were subjected to quality control using fastp [58] prior to mapping to the singleA haplotype genome with hisat2 [59] software. Finally, the TPM (Transcripts Per Kilobase Million) values of the genes were calculated using R packages such as Rsubread, limma, and edgeR [60,61,62]. The resulting data were presented in the form of expression heatmaps using the R statistical computing.

To identify key regulatory genes in the ZZ1 associated with smut resistance, a selection process was initiated where genes with deletions or non-fluctuating data were excluded. Subsequently, Weighted Gene Co-expression Network Analysis (WGCNA) was conducted using the WGCNA package (v1.70-3, https://github.com/nanshanjin/WGCNA (accessed on 18 August 2024)) to identify clusters (modules), of genes exhibiting highly correlated expression patterns. Finally, network maps illustrating these strongly correlated genes were generated using Cytoscape 3.0 [63].

### 5.9. RNA Isolation and RT-qPCR Expression Analysis

Total RNA was extracted from ROC22 (one Sugarcane cultivar) using the RNA prep Pure Plant Kit (DP441) (TIANGEN, Beijing, China) from plant materials including stems, leaves, roots, and buds. Reverse transcription was then performed to generate the required amount of cDNA. For RT-qPCR expression validation, 10% (5 genes) of the 48 GRAS members in the single A subgenome were selected. The selected genes included 4 at random and *ShGRAS21A*, which was of interest in the WGCNA and network analyses, and primers were designed using Primer 5. The experimental template consisted of a 96-well plate, each of which established a 20 μL reaction system. Biological replicates and technical replicates were performed 3 times, the GAPDH gene acting as an internal reference. The relative expression levels of the target genes were determined calculated as follows: 2^−ΔΔCt^ = 2^−(ΔCt treated − ΔCt control)^, where ΔΔCt = ΔCt of the treated sample minus ΔCt of the untreated control sample, and was standardised to buds as control with a value of ‘1’. Results were presented as mean ± standard deviation (SD) from three independent experiments and analyzed by one-way ANOVA with post hoc Tukey’s test.

## Figures and Tables

**Table 1:** The model prediction parameters of the GRAS TFs subfamily.

GRAS Subfamilies	Fraction Disordered	RS	GRAS Subfamilies	Fraction Disordered	RS	RMSD	MatchAlign Score
ShLISCL	0.49	0.82	SbLISCL	0.38	0.83	1.626	1180.0
ShPAT1	0.37	0.82	SbPAT1	0.40	0.81	1.930	1744.5
ShHAM	0.41	0.81	SbHAM	0.40	0.80	2.570	3164.5
ShSHR	0.13	0.91	SbSHR	0.36	0.85	1.524	554.5
ShDELLA	0.49	0.81	SbDELLA	0.30	0.80	28.276	837.0
ShSCR	0.36	0.80	SbSCR	0.17	0.82	0.226	2547.5
ShSCL	0.25	0.84	SbSCL	0.27	0.85	1.201	536.0
ShSCL4/7	0.27	0.82	SbSCL4/7	0.27	0.84	4.022	2794.5
ShLAS	0.24	0.86	SbLAS	0.05	0.89	0.498	1290.0
ShDLT	0.37	0.84	SbDLT	0.39	0.83	0.288	3037.5
ShOs43	0.26	0.84	SbOs43	0.25	0.84	0.568	2655.0

Notes: Fraction disordered: a scalar in the range 0–1 to indicate the proportion of the predicted structure that is disordered. The closer the value is to 1, the higher the proportion of disordered part in the protein structure. Ranking score (RS): a measure of the performance of the prediction algorithm, which evaluates the prediction performance based on the ranking of the node pairs, and a high Ranking Score means good prediction performance. RMSD: Root Mean Square Deviation, is an important measure of the difference between the structures of two molecules or proteins. The smaller the RMSD value, the smaller the difference between the two structures and the more similar the structures. MatchAlign score: a quantitative metric to evaluate the spatial similarity of two protein structures. When the MatchAlign score is higher, there is more structural similarity.

## Data Availability

Data are contained within the article and Supplementary Materials.

## Supplementary Materials

The following supporting information can be downloaded at: https://www.mdpi.com/article/10.3390/ijms252413470/s1.

## References

[1] Karin M. (1990). Too Many Transcription Factors: Positive and Negative Interactions. New Biol..

[2] Latchman D.S. (1993). Transcription Factors: An Overview. Int. J. Exp. Pathol..

[3] Huang W., Xian Z., Kang X., Tang N., Li Z. (2015). Genome-Wide Identification, Phylogeny and Expression Analysis of GRAS Gene Family in Tomato. BMC Plant Biol..

[4] Paz-Ares J., Ghosal D., Wienand U., Peterson P.A., Saedler H. (1987). The Regulatory C1 Locus of Zea Mays Encodes a Protein with Homology to Myb Proto-Oncogene Products and with Structural Similarities to Transcriptional Activators. EMBO J..

[5] Bai Y., Liu H., Zhu K., Cheng Z.-M. (2022). Evolution and Functional Analysis of the GRAS Family Genes in Six Rosaceae Species. BMC Plant Biol..

[6] Fan Y., Yan J., Lai D., Yang H., Xue G., He A., Guo T., Chen L., Cheng X., Xiang D. (2021). Genome-Wide Identification, Expression Analysis, and Functional Study of the GRAS Transcription Factor Family and Its Response to Abiotic Stress in Sorghum [*Sorghum Bicolor* (L.) Moench]. BMC Genom..

[7] Peng J., Carol P., Richards D.E., King K.E., Cowling R.J., Murphy G.P., Harberd N.P. (1997). The *Arabidopsis* GAI Gene Defines a Signaling Pathway That Negatively Regulates Gibberellin Responses. Genes Dev..

[8] Pysh L.D., Wysocka-Diller J.W., Camilleri C., Bouchez D., Benfey P.N. (1999). The GRAS Gene Family in *Arabidopsis*: Sequence Characterization and Basic Expression Analysis of the SCARECROW-LIKE Genes. Plant J..

[9] Rana D., Sharma P., Arpita K., Srivastava H., Sharma S., Gaikwad K. (2023). Genome-Wide Identification and Characterization of GRAS Gene Family in Pigeonpea (*Cajanus cajan* (L.) Millspaugh). 3 Biotech.

[10] Silverstone A.L., Ciampaglio C.N., Sun T. (1998). The *Arabidopsis* RGA Gene Encodes a Transcriptional Regulator Repressing the Gibberellin Signal Transduction Pathway. Plant Cell.

[11] Huang X., Zentella R., Park J., Reser L., Bai D.L., Ross M.M., Shabanowitz J., Hunt D.F., Sun T. (2024). Phosphorylation Activates Master Growth Regulator DELLA by Promoting Histone H2A Binding at Chromatin in Arabidopsis. Nat. Commun..

[12] Hofmann N.R. (2016). A Structure for Plant-Specific Transcription Factors: The GRAS Domain Revealed. Plant Cell.

[13] Liu X., Widmer A., Li S., Zhao Y., Zhao Z., Wu X., Sun L., Liu Q., Wu Y. (2016). Crystal Structure of the GRAS Domain of SCARECROW-LIKE7 in *Oryza Sativa*. Plant Cell.

[14] Hakoshima T. (2018). Structural Basis of the Specific Interactions of GRAS Family Proteins. FEBS Lett..

[15] Liu X., Widmer A. (2014). Genome-Wide Comparative Analysis of the GRAS Gene Family in Populus, *Arabidopsis* and Rice. Plant Mol. Biol. Report..

[16] Tian C., Wan P., Sun S., Li J., Chen M. (2004). Genome-Wide Analysis of the GRAS Gene Family in Rice and *Arabidopsis*. Plant Mol. Biol..

[17] Hirsch S., Kim J., Muñoz A., Heckmann A.B., Downie J.A., Oldroyd G.E.D. (2009). GRAS Proteins Form a DNA Binding Complex to Induce Gene Expression during Nodulation Signaling in *Medicago truncatula*. Nat. Genet..

[18] To V.-T., Shi Q., Zhang Y., Shi J., Shen C., Zhang D., Cai W. (2020). Genome-Wide Analysis of the GRAS Gene Family in Barley (*Hordeum Vulgare* L.). Genes.

[19] Song X.-M., Liu T.-K., Duan W.-K., Ma Q.-H., Ren J., Wang Z., Li Y., Hou X.-L. (2014). Genome-Wide Analysis of the GRAS Gene Family in Chinese Cabbage (*Brassica rapa* ssp. Pekinensis). Genomics.

[20] Zhao X., Xu Y., He G., He K., Xiao L., Hu R., Li S. (2022). Genome-Wide Characterization and Expression Profiling of the GRAS Gene Family in Salt and Alkali Stresses in *Miscanthus Sinensis*. Int. J. Mol. Sci..

[21] Tong N., Li D., Zhang S., Tang M., Chen Y., Zhang Z., Huang Y., Lin Y., Cheng Z., Lai Z. (2023). Genome-Wide Identification and Expression Analysis of the GRAS Family under Low-Temperature Stress in Bananas. Front. Plant Sci..

[22] Zaman Q.U., Hussain M.A., Khan L.U., Cui J.-P., Hui L., Khan D., Lv W., Wang H.-F. (2022). Genome-Wide Identification and Expression Pattern of the GRAS Gene Family in Pitaya (*Selenicereus undatus* L.). Biology.

[23] Zhang H., Mi L., Xu L., Yu C., Li C., Chen C. (2019). Genome-Wide Identification, Characterization, Interaction Network and Expression Profile of GRAS Gene Family in Sweet Orange (*Citrus sinensis*). Sci. Rep..

[24] Kumar B., Bhalothia P., Chen J., Yan Q., Li J., Feng L., Zhang Y., Xu J., Xia R., Zeng Z. (2021). The GRAS Gene Family and Its Roles in Seed Development in Litchi (*Litchi chinensis* Sonn). BMC Plant Biol..

[25] Jing C.-Y., Zhang F.-M., Wang X.-H., Wang M.-X., Zhou L., Cai Z., Han J.-D., Geng M.-F., Yu W.-H., Jiao Z.-H. (2022). Genomic Insights into the Recent Chromosome Reduction of Autopolyploid Sugarcane *Saccharum spontaneum*. Nat. Genet..

[26] Yu F., Wang P., Li X., Huang Y., Wang Q., Luo L., Jing Y., Liu X., Deng Z., Wu J. (2018). Characterization of Chromosome Composition of Sugarcane in Nobilization by Using Genomic in Situ Hybridization. Mol. Cytogenet..

[27] Bao Y., Zhang Q., Huang J., Zhang S., Yao W., Yu Z., Deng Z., Yu J., Kong W., Yu X. (2024). A Chromosomal-Scale Genome Assembly of Modern Cultivated Hybrid Sugarcane Provides Insights into Origination and Evolution. Nat. Commun..

[28] Cenci A., Rouard M. (2017). Evolutionary Analyses of GRAS Transcription Factors in *Angiosperms*. Front. Plant Sci..

[29] Liu B., Sun Y., Xue J., Jia X., Li R. (2018). Genome-Wide Characterization and Expression Analysis of GRAS Gene Family in Pepper (*Capsicum Annuum* L.). PeerJ.

[30] Wang X., Li G., Sun Y., Qin Z., Feng P. (2021). Genome-Wide Analysis and Characterization of GRAS Family in Switchgrass. Bioengineered.

[31] Sun X., Jones W.T., Rikkerink E.H.A. (2012). GRAS Proteins: The Versatile Roles of Intrinsically Disordered Proteins in Plant Signalling. Biochem. J..

[32] Dobrev V.S., Fred L.M., Gerhart K.P., Metallo S.J. (2018). Characterization of the Binding of Small Molecules to Intrinsically Disordered Proteins. Methods in Enzymology.

[33] Lee R., van der Buljan M., Lang B., Weatheritt R.J., Daughdrill G.W., Dunker A.K., Fuxreiter M., Gough J., Gsponer J., Jones D.T. Classification of Intrinsically Disordered Regions and Proteins. https://pubs.acs.org/doi/full/10.1021/cr400525m.

[34] Sun X., Xue B., Jones W.T., Rikkerink E., Dunker A.K., Uversky V.N. (2011). A Functionally Required Unfoldome from the Plant Kingdom: Intrinsically Disordered N-Terminal Domains of GRAS Proteins Are Involved in Molecular Recognition during Plant Development. Plant Mol. Biol..

[35] Lu L., Diao Z., Yang D., Wang X., Zheng X., Xiang X., Xiao Y., Chen Z., Wang W., Wu Y. (2022). The 14-3-3 Protein GF14c Positively Regulates Immunity by Modulating the Protein Homoeostasis of the GRAS Protein *OsSCL7* in Rice. Plant Cell Environ..

[36] De Vleesschauwer D., Seifi H.S., Filipe O., Haeck A., Huu S.N., Demeestere K., Höfte M. (2016). The DELLA Protein SLR1 Integrates and Amplifies Salicylic Acid- and Jasmonic Acid-Dependent Innate Immunity in Rice. Plant Physiol..

[37] Hou J., Xiao H., Yao P., Ma X., Shi Q., Yang J., Hou H., Li L. (2024). Unveiling the Mechanism of Broad-spectrum Blast Resistance in Rice: The Collaborative Role of Transcription Factor OsGRAS30 and Histone Deacetylase *OsHDAC1*. Plant Biotechnol. J..

[38] Gullerova M., Barta A., Lorković Z.J. (2006). AtCyp59 Is a Multidomain Cyclophilin from *Arabidopsis thaliana* That Interacts with SR Proteins and the C-Terminal Domain of the RNA Polymerase II. RNA.

[39] Sharma A., Sharma D., Verma S.K. (2019). Zinc Binding Proteome of a Phytopathogen *Xanthomonas Translucens Pv. Undulosa*. Plant Physiol. Biochem..

[40] Sharma A., Sharma D., Verma S.K., Saha D., Prasad A.M., Srinivasan R. (2007). Pentatricopeptide Repeat Proteins and Their Emerging Roles in Plants. Plant Physiol. Biochem..

[41] Zeiner A., Colina F.J., Citterico M., Wrzaczek M. (2023). CYSTEINE-RICH RECEPTOR-LIKE PROTEIN KINASES: Their Evolution, Structure, and Roles in Stress Response and Development. J. Exp. Bot..

[42] Craig A., Ewan R., Mesmar J., Gudipati V., Sadanandom A. (2009). E3 Ubiquitin Ligases and Plant Innate Immunity. J. Exp. Bot..

[43] Snedden W.A., Fromm H. (1998). Calmodulin, Calmodulin-Related Proteins and Plant Responses to the Environment. Trends Plant Sci..

[44] Vij S., Tyagi A.K. (2008). A20/AN1 Zinc-Finger Domain-Containing Proteins in Plants and Animals Represent Common Elements in Stress Response. Funct. Integr. Genom..

[45] Wani S.H., Anand S., Singh B., Bohra A., Joshi R. (2021). WRKY Transcription Factors and Plant Defense Responses: Latest Discoveries and Future Prospects. Plant Cell Rep..

[46] Finn R.D., Clements J., Eddy S.R. (2011). HMMER Web Server: Interactive Sequence Similarity Searching. Nucleic Acids Res..

[47] Altschul S.F., Gish W., Miller W., Myers E.W., Lipman D.J. (1990). Basic Local Alignment Search Tool. J. Mol. Biol..

[48] Gel B., Serra E. (2017). karyoploteR: An R/Bioconductor Package to Plot Customizable Genomes Displaying Arbitrary Data. Bioinformatics.

[49] Osorio D., Rondón-Villarreal P., Torres R. (2015). Peptides: A Package for Data Mining of Antimicrobial Peptides. R J..

[50] Edgar R.C., Li W.-Y., Wang X., Li R., Li W.-Q., Chen K.-M. (2004). MUSCLE: Multiple Sequence Alignment with High Accuracy and High Throughput. Nucleic Acids Res..

[51] Stamatakis A. (2014). RAxML Version 8: A Tool for Phylogenetic Analysis and Post-Analysis of Large Phylogenies. Bioinformatics.

[52] Dutta M., Saha A., Moin M., Kirti P.B. (2021). Genome-Wide Identification, Transcript Profiling and Bioinformatic Analyses of GRAS Transcription Factor Genes in Rice. Front. Plant Sci..

[53] Chen C., Chen H., Zhang Y., Thomas H.R., Frank M.H., He Y., Xia R. (2020). TBtools: An Integrative Toolkit Developed for Interactive Analyses of Big Biological Data. Mol. Plant.

[54] Bailey T.L., Johnson J., Grant C.E., Noble W.S. (2015). The MEME Suite. Nucleic Acids Res..

[55] Lescot M. (2002). PlantCARE, a Database of Plant Cis-Acting Regulatory Elements and a Portal to Tools for in Silico Analysis of Promoter Sequences. Nucleic Acids Res..

[56] Abramson J., Adler J., Dunger J., Evans R., Green T., Pritzel A., Ronneberger O., Willmore L., Ballard A.J., Bambrick J. (2024). Accurate Structure Prediction of Biomolecular Interactions with AlphaFold 3. Nature.

[57] Bramucci E., Paiardini A., Bossa F., Pascarella S. (2012). PyMod: Sequence Similarity Searches, Multiple Sequence-Structure Alignments, and Homology Modeling within PyMOL. BMC Bioinform..

[58] Chen S., Zhou Y., Chen Y., Gu J. (2018). Fastp: An Ultra-Fast All-in-One FASTQ Preprocessor. Bioinformatics.

[59] Zhang Y., Park C., Bennett C., Thornton M., Kim D. (2021). Rapid and Accurate Alignment of Nucleotide Conversion Sequencing Reads with HISAT-3N. Genome Res..

[60] Chen Y., Chen L., Lun A.T.L., Baldoni P.L., Smyth G.K. (2024). edgeR 4.0: Powerful Differential Analysis of Sequencing Data with Expanded Functionality and Improved Support for Small Counts and Larger Datasets. bioRxiv.

[61] Liao Y., Smyth G.K., Shi W. (2019). The R Package Rsubread Is Easier, Faster, Cheaper and Better for Alignment and Quantification of RNA Sequencing Reads. Nucleic Acids Res..

[62] Ritchie M.E., Phipson B., Wu D., Hu Y., Law C.W., Shi W., Smyth G.K. (2015). Limma Powers Differential Expression Analyses for RNA-Sequencing and Microarray Studies. Nucleic Acids Res..

[63] Shannon P., Markiel A., Ozier O., Baliga N.S., Wang J.T., Ramage D., Amin N., Schwikowski B., Ideker T. (2003). Cytoscape: A Software Environment for Integrated Models of Biomolecular Interaction Networks. Genome Res..

