# Supplementary material for: Genome-Wide Identification and Characterization of GRAS Transcription Factor Family in Cultivated Hybrid Sugarcane ZZ1 (Saccharum officinarum) and Their Role in Development and Stress
Source: Int J Mol Sci. 2024 Dec 16;25(24):13470. doi: 10.3390/ijms252413470 (PMC11678106; doi:10.3390/ijms252413470)
Supplement: Supplementary file 1 [file ijms-25-13470-s001.zip › Figure Supplementary.pdf]

Genome-Wide Identification and Characterization of GRAS Transcription Factor Family in Cultivated Hybrid Sugarcane ZZ1 (*Saccharum officinarum*) and Their Role in Development and Stress

Hao Wen [1], Lidan Wang [2], Yuqing Gong [3], Yu Zhang [3], Tingting Zhao [1], Cuilian Feng [1], Jungang Wang [1,*] and Jishan Lin [1,*]

[1] National Key Laboratory for Tropical Crop Breeding, Institute of Tropical Bioscience and Biotechnology, Chinese Academy of Tropical Agricultural Sciences, Sanya 572024, China; wenhaohy34@163.com (H.W.); zhaotingting@itbb.org.cn (T.Z.); fengcuilian@itbb.org.cn (C.F.)

[2] School of Breeding and Multiplication (Sanya Institute of Breeding and Multiplication), Hainan University, Sanya 572025, China; 15528556909@163.com

[3] Haixia Institute of Science and Technology, Fujian Agriculture and Forestry University,
Fuzhou 350002, China; gongyuqing497@163.com (Y.G.); 18055789001@163.com (Y.Z.)

* Correspondence: wangjungang@itbb.org.cn (J.W.); linjishan@itbb.org.cn (J.L.)

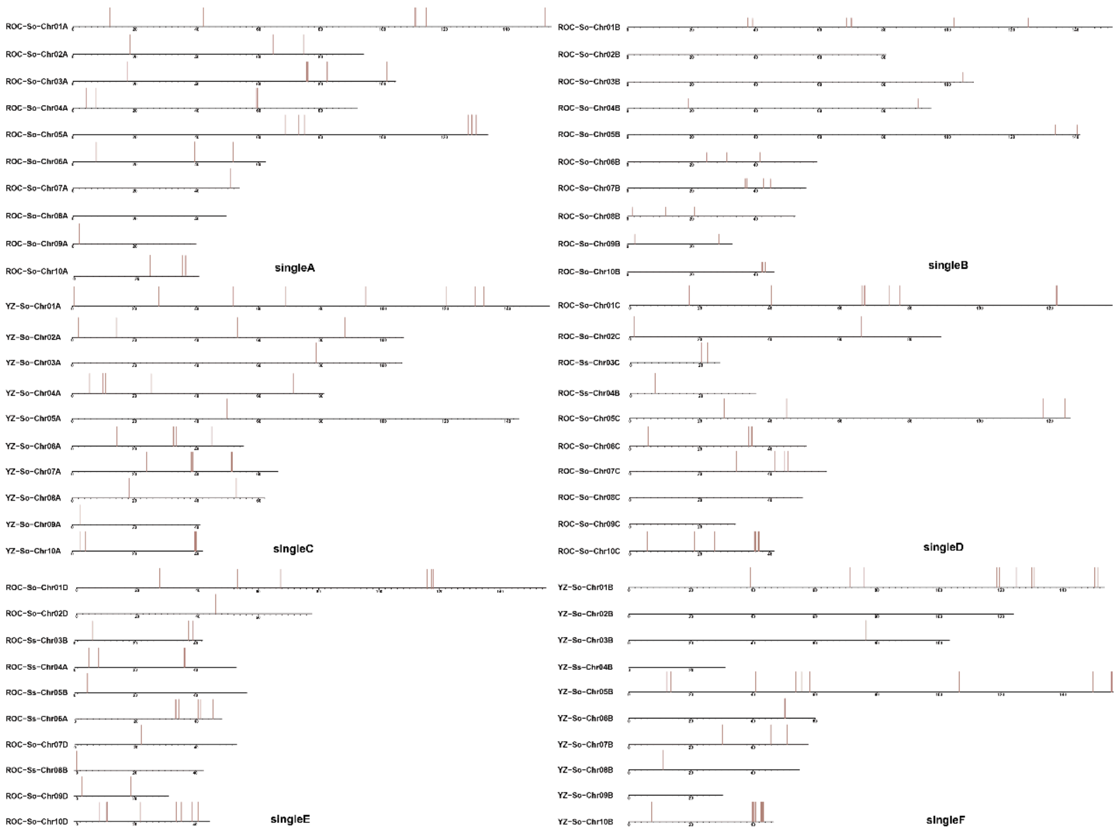

Figure S1. Physical location of the GRAS gene of Modern sugarcane hybrids (ZZ1) on subgenomic singleA to singleF chromosomes.

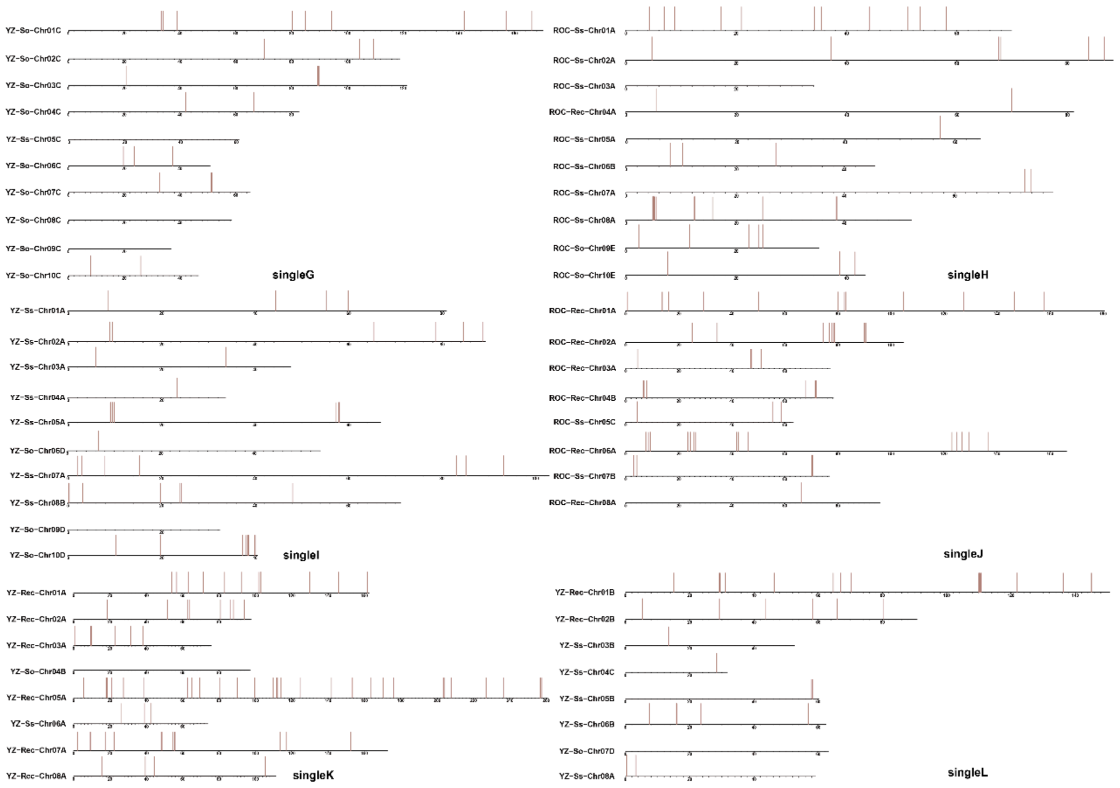

Figure S2. Physical location of the GRAS gene of Modern sugarcane hybrids (ZZ1) on subgenomic

singleG to singleL chromosomes.

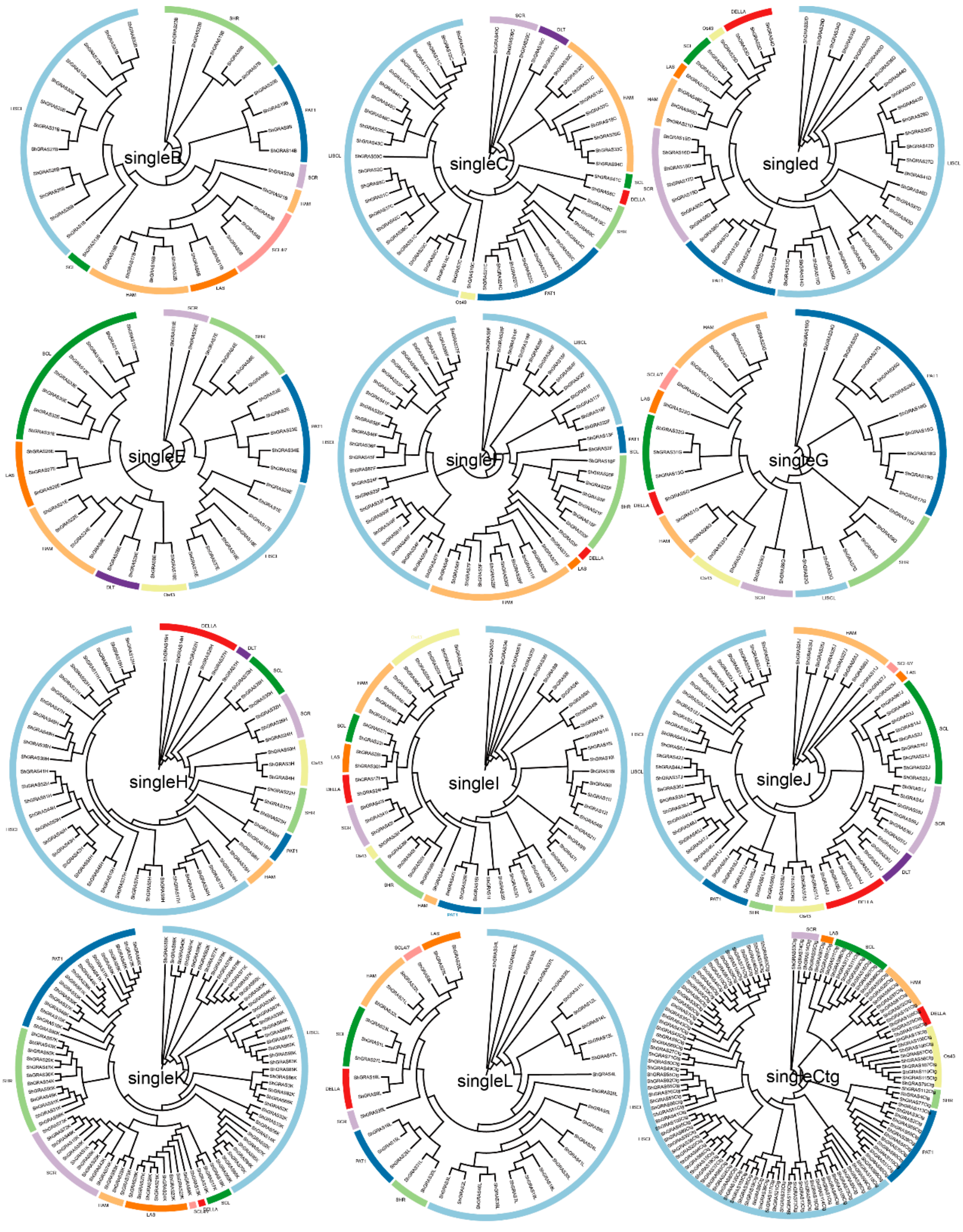

Figure S3. Phylogenetic tree of the GRAS gene of Modern sugarcane hybrids (ZZ1) in subgenomes singleB to singleCtg.

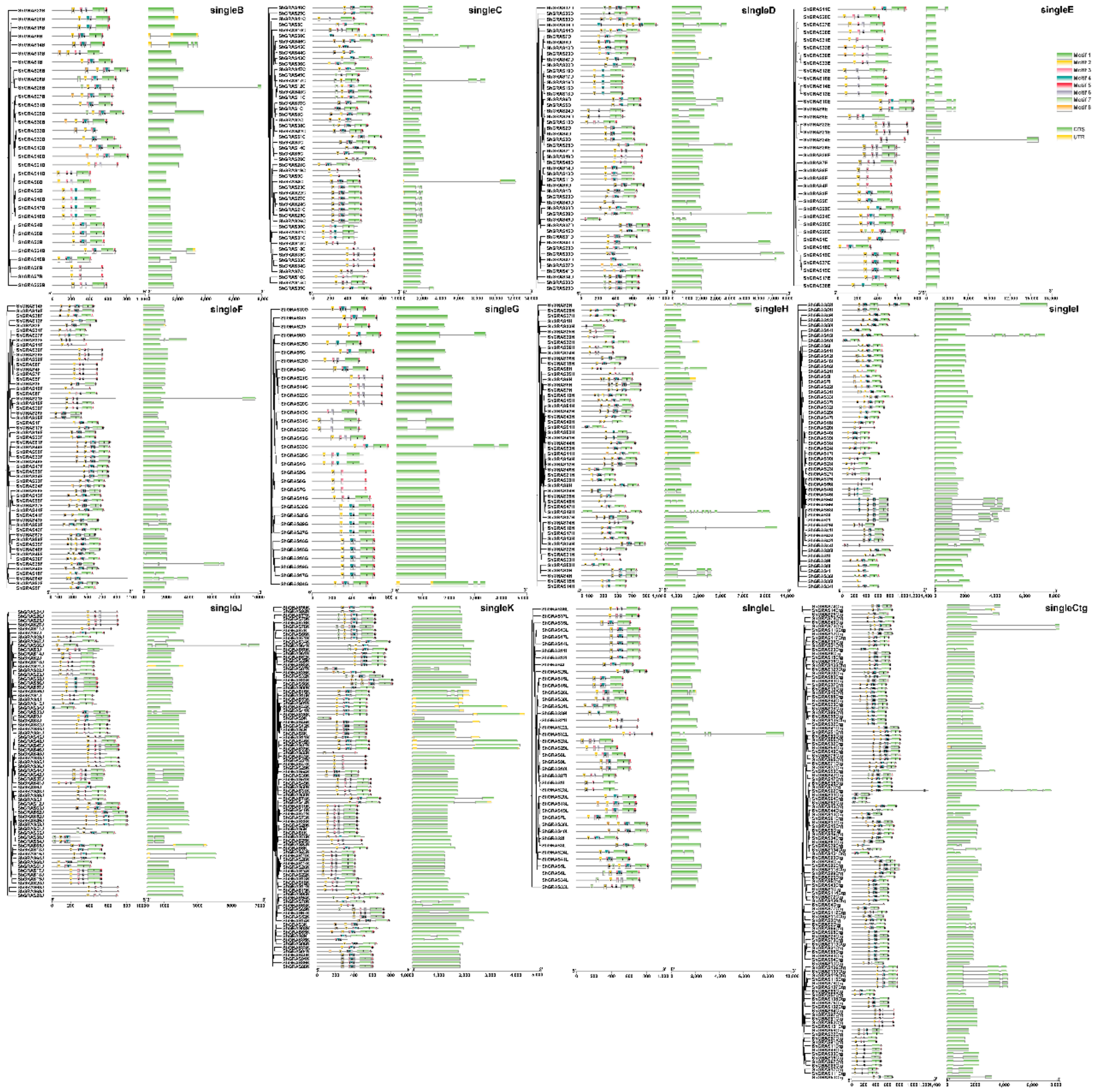

Figure S4. Phylogenetic relationships, motif distributions and gene structure of GRAS genes in Modern sugarcane hybrids (ZZ1) subgenomes singleB to singleCtg.
